# Is the Cost per Occupied Bed the Only Criterion of Efficency and Economy?

**Published:** 1920-01-10

**Authors:** 


### THE KING EDWARD'S FUND STATISTICAL REPORT.

### III. Is the Cost per Occupied Bed the Only Criterion of Efficency and Economy?

To continue our survey of the report before proceeding to other matters it is remarked that in the case of the smaller general hospitals, the consumption hospitals, hospitals for women, and ophthalmic hospitals, there is little diversification in cost. The averages of the children's hospitals run chiefly between £105 and £120 per occupied bed, except the Hospital for Sick Children, which is again the lowest with a cost of £91 16s. 1d. The large nerve disease hospital is compared with the two smaller ones with results that are not unexpected.

In the section supplying notes from hospital authorities, which are intended to explain statistical information given elsewhere, it is evident that many of the notes have not been recently revised by the hospital secretaries. For instance, one states that as there are no paying probationers there is no fee to reduce nursing costs. As the secretary must very well know, even if there were a fee it would appear on the income side of the accounts and would not, under existing rules, be deducted from the nursing salaries. The same hospital says that a larger number of nurses are necessary on the staff of a hospital without a medical school because they have to do the work done by students in hospitals with medical schools. It is, however, our experience that hospitals with medical schools are not less costly in the matter of nursing salaries than other hospitals.

### How is the Money Spent?

Our chief quarrel with the King's Fund Statistics is that everything is translated into terms of cost per occupied bed, and cost per out-patient attendance, thus cultivating a narrow view of the functions of a voluntary hospital. It is obvious that it is not extravagance or mismanagement alone that causes the extraordinary differences in in-patient cost revealed by the statistics before us. The difference is no less remarkable when the figures are arranged by another method, in the table printed on this page, where it is shown that although seven of the hospitals quoted maintain about the same number of beds for each £10,000, the number of out-patient attendances covered at the same time varies from 18,000 to 34,000, while in the extreme cases the divergence is more in beds (40 against 84), than in the out-patient attendances (18,000 against 19,000). The clue to the position is unquestionably to be decided by two factors: (1) What is considered suitable expense, and (2) what is achieved in matters which are not entirely a question of treatment of patients at all. Taking these two points together we are sure that could the matter be sifted to the utmost, it would be found that divergence in cost means very largely divergence in size, quality, and cost of staff and very little in prices paid for food and other supplies.

### The Unknown Factor.

When, therefore, we come to examine the figures of different hospitals side by side, before coming to any conclusion it is necessary to know far more than the statistical report is able to tell us. We need to be told what is the scientific work carried out at each hospital, we must visit the laboratory and the lecture room, we must be informed as to the extent and quality of the arrangements for the in-patient and out-patient surgical work, we must be assured as to the *x*-ray and light, dental, and other departments. Then we shall have to know more as to the size of the nursing staff, the hours of duty of the nurses, their pay, the thoroughness of their training and their after-success in their profession. We must be told whether the patients are let loose into the work-a-day world half convalescent or not at all convalescent, to take a haphazard chance as to whether the infinite pains that have been given on their behalf may not in many cases be entirely thrown away. We must know, in short, whether there is a properly organised after-care or almoner's department to secure that the greatest value shall be obtained from what the public has provided and to secure what is the greatest economy of all: permanent benefit to the patient.

Table Showing how the Larger General Hospitals, with Medical Schools, Spend Each £10,000 Entrusted to Them.

| Hospital | Total expenditure (nearest £1,000) | Occupied Beds | Out-patient attendances (nearest 1,000) | Cost per occupied bed (nearest £1) | Cost per out-patient attendance (nearest penny) | What £10,000 pays for: Bed maintenance (nearest unit) | O.P. attendance (nearest 1,000) | Other purposes |
|---|---|---|---|---|---|---|---|---|
| Charing Cross ... | 44,000 | 250 | 81.000 | 124 | 19 | 56 | 18.000 | A value, entirely undisclosed by the Statistical Report, as regards Scientific Research, Training of Nurses, After-Care or Almoner's Work, and other matters. |
| Guy's ... ... | 109.000 | 539 | 376,000 | 132 | 12 | 49 | 34,000 | |
| London ... ... | 200 000 | 805 | 356,000 | 175 | 21 | 40 | 18,0 0 | |
| Middlesex ... ... | 53 000 | 291 | 166,000 | 131 | 11 | 55 | 31.000 | |
| Royal Free ... ... | 36,000 | 174 | 121,000 | 133 | 19 | 48 | 34.000 | |
| St. George's ... | 59 000 | 300 | 111.000 | 153 | 14 | 51 | 19.000 | |
| St. Mary's ... ... | 46,000 | 251 | 96,000 | 137 | 19 | 55 | 21,000 | |
| University College | 55.000 | 4 0 | 135 000 | 86 | 16 | 84 | 25 000 | |
| Westminster ... | 35,000 | 192 | 71,000 | 141 | 13 | 55 | 20,000 | |

Statistics of King's College Hospital and St. Thomas's Hospital, because of the arrangement of the military section, are not available for comparison in 1918.

